# Statistical learning as a window into developmental disabilities

**DOI:** 10.1186/s11689-018-9252-y

**Published:** 2018-12-13

**Authors:** Jenny R. Saffran

**Affiliations:** Waisman Center, University of Wisconsin—Madison, 1500 Highland Ave, Madison, WI 53705 USA

**Keywords:** Learning, Language, Speech, Autism, Developmental language disorder, Williams syndrome

## Abstract

Until recently, most behavioral studies of children with intellectual and developmental disabilities (IDD) have used standardized assessments as a means to probe etiology and to characterize phenotypes. Over the past decade, however, tasks originally developed to investigate learning processes in typical development have been brought to bear on developmental processes in children with IDD.

This brief review will focus on one learning process in particular—statistical learning—and will provide an overview of what has been learned thus far from studies using statistical learning tasks with different groups of children with IDD conditions. While a full picture is not yet available, results to date suggest that studies of learning are both feasible and informative about learning processes that may differ across diagnostic groups, particularly as they relate to language acquisition.

More generally, studies focused on learning processes may be highly informative about different developmental trajectories both across groups and within groups of children.

## Background

Language is arguably the most complex system that humans have to learn. Unlike other complex learning problems like reading or calculus, language learning primarily takes place during the first few years of life. This pair of observations suggests that the brains of infants and young children must be especially well-suited to acquiring the structures of natural languages. Indeed, language-learning abilities decline after early childhood [1, 2], in contrast to most other cognitive skills, which increase over the course of childhood.

This state of affairs—highly efficient and effective learning of a vastly complex system by very young brains—is a fascinating scientific situation in the case of typical development: children somehow discern the myriad complexities of their native language—or languages—with impressive rapidity and in the absence of explicit teaching. These linguistic processes range from discovering native language phonemes to discerning which sound sequences map onto meanings to organizing words into categories and sequencing those categories into syntactic structures. For children with developmental disabilities, however, language acquisition is an area of significant concern. Challenges in language learning are common to most forms of intellectual disability. Of particular interest is the fact that these challenges take different forms for different groups of children. Even within a single diagnostic group, there is a great deal of variation in outcomes.

Until recently, research on language acquisition in children with intellectual and developmental disabilities (IDD) largely centered around the use of standardized assessments. These include measures of children’s language attainments gathered from direct testing or observation of the child and measures taken via parental report. In recent years, however, this literature has been augmented by studies focused on learning itself. By providing infants and children with carefully designed learning tasks, and assessing the knowledge that is acquired (via behavioral and/or neuroimaging methods), researchers have been able to go beyond descriptions of language attainments to describe the underlying mechanisms that may have led to those attainments. This focus on learning is significant because different learning trajectories—in terms of learning mechanisms themselves, or the perceptual or cognitive structures of which learning operates—may help to explain the varied patterns of language outcomes that are observed in groups of individuals with IDDs.

## Statistical learning studies in children with IDDs

One aspect of language acquisition that has been of particular interest in this regard is statistical learning, a form of implicit learning that entails detecting patterns in the input (for a recent review, see 3). Statistical learning abilities in human infants have been described across numerous domains of functioning, ranging from language to music to vision to action. They have been implicated in infants’ discovery of categories, including native language phonemes or lexical categories [4], discovery of concrete units, such as words in fluent speech [5, 6], and discovery of structure, as in linguistic phrase structure [7]. While these abilities appear to be quite general in the sense that they operate over a range of different types of input, they are constrained both by the types of input they track and the modalities in which those inputs appear [3, 4, 8]. For example, infants appear to have more difficulty tracking sequential statistical patterns in visual stimuli than in auditory stimuli [9–11].

Statistical learning abilities exhibit substantial individual variation. Some of these differences may reflect measurement issues; the tasks used to assess statistical learning in children and adults often entail forced-choice tests or other judgment tasks that tap explicit representations of implicitly acquired knowledge (though see [12–14] for examples of tasks that use implicit learning measures with adults). That is, it is difficult to determine, from a forced-choice test with substantial working memory demands, whether scores veridically reflect learning outcomes, or whether scores are also influenced by other factors (e.g., metalinguistic awareness, cognitive control, attention) Nevertheless, performance on statistical language learning tasks is correlated with native language attainment and second language learning in adults, at least in some studies [15–17]. Performance in nonlinguistic statistical learning tasks is also correlated with native language processing [18], though there is also evidence for domain-specific relationships [19]. Similar relationships between statistical learning and native language processing have been observed in samples of typically developing children and infants [20–24]. To the extent that individual differences hold up under further empirical scrutiny, they are consistent with the hypothesis that statistical learning processes are entailed in native language acquisition.

For researchers and clinicians interested in language disorders and other forms of IDD, comparisons between groups of infants or children who are known to be following different developmental trajectories are of particular interest. In particular, can patterns of strengths and weaknesses in statistical learning help to explain the patterns of deficits in IDD? The first such study used a statistical learning task to test adolescents with Specific language impairment (SLI), a disability characterized by relative weakness in native language abilities in contrast to age-appropriate performance on measures of other cognitive skills [25] (note that SLI is now referred to as Developmental Language Disorder, or DLD [26]; for the purpose of this review, we will use the diagnostic terminology used at the time this research was conducted). Adolescents with SLI—especially those with grammatical impairments—showed relatively weak performance on a serial reaction time task assessing visual statistical learning. Of note, this task involved tracking patterns of nonlinguistic shapes, suggesting that the learning challenges these children experience are not limited to either linguistic nor auditory stimuli.

In a study by Evans et al. [27], grade-school-aged children diagnosed with SLI performed a linguistic statistical learning task in which they listened to streams of syllables organized into words, with only statistical cues to word boundaries. The children with SLI were compared with a control group of typically developing children matched on chronological age and nonverbal IQ. As in Tomblin et al.’s [25] study with visual materials, the children with SLI performed more poorly than matched controls on the statistical language learning task, requiring more time to show evidence of learning on this task (see [28] for similar results from adults with DLD). Performance was even worse on a non-linguistic version of the statistical learning task in which sequences were created from musical tones, again supporting the view that the challenges these children face are not limited to linguistic stimuli.

A recent meta-analysis of the extant literature confirmed this general pattern: children with SLI do more poorly on statistical language learning tasks than children who are typical language learners [29]. These data suggest that consistent with the results of Tomblin et al. [25], children with SLI exhibit difficulty in tracking sequential patterns that are both linguistic and nonlinguistic, supporting the view that the deficits observed in SLI are not specific to language [2, 30]. This general pattern of results is intriguing given the potential links between performance on statistical learning tasks and native language attainment that have been observed in the literature [20–24]. Moreover, a recent study suggested a similar conclusion based on comparisons between children with developmental dyslexia (DD) and children with typical development [31]. Children with DD did more poorly on linguistic and tone-sequence statistical learning tasks than children in the comparison group. Moreover, performance on both the linguistic and nonlinguistic statistical learning tasks was correlated with reading measures. These data are consistent with the view that challenges in learning underlie at least some aspects of both SLI and developmental dyslexia, an argument based on a meta-analysis of studies using serial reaction time measures of procedural learning [32]. That said, it seems likely that given the complexity of these behavioral phenotypes, and the range of outcomes with which they are associated, there are likely a number of cognitive and/or perceptual deficits that work together as these disabilities develop [33, 34].

It is not the case, though, that all developmental language learning deficits can be attributed to challenges in statistical learning. The meta-analysis of the SLI statistical language learning literature described above also examined the extant literature on statistical learning in children and adolescents with autism spectrum disorders [29]. Strikingly, the data across numerous studies suggest that autistic individuals do not show difficulties in statistical learning tasks. For example, Mayo and Eigsti [35] tested autistic children with a history of language delay using the same materials previously used by Evans et al. [27] in their study of children with SLI: a stream of syllables, presented continuously, containing trisyllabic “words” with only statistical cues to word boundaries. The question of interest was whether children would be able to correctly distinguish words from sequences that were not words based solely on their statistical properties. The autistic children showed the same pattern of performance as children with typical development, successfully selecting words on the forced-choice test trials. These data suggest that not all language and developmental disorders include challenges with statistical learning. Similar results were observed for a visual statistical learning task, with children with ASD performing comparably to children with typical development [36]. Interestingly, the same study found that adults with ASD outperformed adults in the comparison group, suggesting that enhanced processing of visual details in individuals with ASD may in fact facilitate statistical learning.

In a direct comparison between autism and SLI, Haebig et al. [37] tested grade-school-aged children diagnosed with one of these IDD conditions on a statistical language learning task, again involving discovery of words in fluent speech based on their statistical properties. Using identical methods and materials with the two groups of children, they observed a divergence in performance, such that children with autism outperformed children with SLI. The degree of language impairment within the group of children with autism did not predict performance in this linguistic task, consistent with the view that verbal statistical learning, at least, is relatively robust in this population of children.

That said, this study, along with the vast majority of studies investigating language learning in autistic children, sampled relatively high functioning children in tasks with explicit testing formats. Haebig et al. [37] only included only children who could perform this rather challenging task (which involved listening to streams of syllables and making judgements about which sequences were more word-like in a forced-choice task). Autistic children who show more language deficits, or greater severity of other behavioral symptoms typically associated with autism, may exhibit different patterns of functioning, performing less well on statistical learning tasks. Moreover, studies that have employed neuroimaging methods rather than behavioral methods have uncovered different patterns of results. For example, Scott-Van Zeeland et al. [38] found no evidence of sensitivity to statistical regularities in an fMRI task with autistic children. In a study using a mismatch paradigm with EEG, Jeste et al. [39] observed different neural reactions in a visual statistical learning task for children with ASD and children with typical development. In particular, children with ASD who had higher non-verbal IQ scores showed a larger brain response to unexpected sequences, whereas children with typical development showed a greater response to the expected trials, suggesting greater allocation of attention to unexpected events. The authors concluded that even within the group of children with ASD, there was a range of neural responses that merit further exploration. In general, it appears that the use of more sensitive test methods that do not require explicit judgements (as in forced-choice tasks) may provide a better index of individual differences in the IDD population. By doing so, the relationship between language development outcomes and statistical learning abilities will become clearer. Limits in populations tested (high-functioning children) and methods (explicit tests with metalinguistic demands) currently constrain our ability to theorize in this regard.

A different approach to understanding the relationship between learning and IDD entails testing infants. Infant studies permit clearer dissociation between the starting state and the experiences and interventions that children have experienced, in order to draw connections between diagnoses and outcomes. Diagnoses like SLI, DD, and autism cannot be made in infancy, and to date, there are no infant high-risk sibling studies of learning (statistical learning or otherwise). However, other types of developmental disorders, such as genetic syndromes arising from deletions or point mutations, are diagnosed in infancy and provide a fascinating opportunity to examine early learning abilities.

Williams Syndrome (WS) is a genetic disorder that is associated with significant intellectual disabilities (especially in the visuospatial domain), paired with relative sparing of language abilities. While the onset of language is often delayed in this group, their early expressive vocabularies outstrip those of children with other IDDs, such as Down syndrome [40]. In a recent study, Cashon et al. [41] tested a group of infants with WS (8- to 20-month-olds) on the same artificial language segmentation task used by Saffran et al. [6]. Infants showed the same pattern of performance observed in studies of typically developing infants. These data provide the first evidence that infants with developmental disorders can track sequential statistics.

How these abilities are used by infants and young children—and the manner in which detection of statistical regularities is combined with detection of other patterns in language—may help us to understand the developmental trajectories characterizing children with different disorders and individual differences more generally [42]. For example, infants with WS may be more reliant on prosodic cues (pitch and rhythmic patterns in language) than their typically developing peers [43]. Studies that focus not just on a single set of cues, but that include more complex and ecologically valid materials, are likely to be highly informative as this field moves forward.

## Conclusions

Emerging theories pertaining to the core deficits observed in different IDD are also integrating considerations of learning in new and exciting ways. For example, several different research groups have recently posited prediction deficit accounts of ASD [44–46]. Predictive processes have been implicated in multiple domains of cognition and behavior [47]. Prediction has also been implicated as an important component of early learning, with violations of predictions serving as error signals, prompting increased attention and cognitive change [48–50]. To the extent that predictive processes are disrupted in autism or other IDD conditions, learning may be impacted. For example, one current hypothesis suggests that autism may be associated with hyperplasticity, such that autistic individuals essentially overweight the most recent events rather than maintaining a veridical representation of the world [45]. On this view, autistic people may track statistics just as neurotypical people do when the environment is stable. But when change occurs, as it so often does, they may have more difficulty integrating new events with prior experiences. This hypothesis provides a potential explanation for why studies of statistical learning in autistic participants have not shown any differences from neurotypical participants: only stable statistical distributions have ever been tested. Learning differences may be more likely to emerge when the learning task involves changing distributions of events.

Research linking learning developmental challenges to different brain areas is also likely to be quite informative as this literature continues to develop, including both cortical and subcortical circuitry [51]. Teasing apart the brain systems that are linked to different early learning processes in typical development provides potential insights into where learning mechanisms might diverge in children with IDD. To the extent that IDD conditions implicate the brain and cognitive systems subserving learning, we can expect new theories to emerge, with implications for novel intervention strategies focused not just on what children know, but how learning itself unfolds.
